# AOPxSVM: A Support Vector Machine for Identifying Antioxidant Peptides Using a Block Substitution Matrix and Amino Acid Composition, Transformation, and Distribution Embeddings

**DOI:** 10.3390/foods14122014

**Published:** 2025-06-06

**Authors:** Rujun Li, Haotian Wang, Qiunan Yu, Jing Cai, Liangzhen Jiang, Ximei Luo, Quan Zou, Zhibin Lv

**Affiliations:** 1 College of Biomedical Engineering, Sichuan University, Chengdu 610041, China; lirujun3303@foxmail.com (R.L.); wanghaotian70094@foxmail.com (H.W.); yuqiunan1234@foxmail.com (Q.Y.); caijing1128@foxmail.com (J.C.); 2 College of Food and Biological Engineering, Chengdu University, Chengdu 610106, China; jiangliangzhen@cdu.edu.cn; 3 Country Key Laboratory of Coarse Cereal Processing, Ministry of Agriculture and Rural Affairs, Chengdu 610106, China; 4 Institute of Fundamental and Frontier Sciences, University of Electronic Science and Technology, Chengdu 610106, China; luoximei@uestc.edu.cn (X.L.); zouquan@nclab.net (Q.Z.)

**Keywords:** antioxidant peptide identification, machine learning, feature engineering optimization, SVM, LGBM

## Abstract

Antioxidant peptides (AOPs) have the natural properties of food preservatives; they are capable of improving the oxidation stability of food while also providing additional benefits such as disease prevention. Traditional experimental methods for identifying antioxidant peptides are time consuming and costly, so effective machine learning models are increasingly being valued by researchers. In this study, we integrated amino acid composition, transformation, and distribution (CTD) and block substitution matrix 62 (BLOSUM62) to develop an SVM-based AOP prediction model called AOPxSVM. This strategy significantly improves the prediction accuracy of the model by comparing 15 feature combinations and feature selection strategies, with their effectiveness being visually verified using UMAP. AOPxSVM achieves high accuracy values of 0.9092 and 0.9330, as well as Matthew’s correlation coefficients (MCCs) of 0.8253 and 0.8670, on two independent test sets, both surpassing the state-of-the-art methods based on the same test sets, thus demonstrating AOPs’ excellent identification capability. We believe that AOPxSVM can serve as a powerful tool for identifying AOPs.

## 1. Introduction

Antioxidants play a critical role in the food industry due to their ability to counteract oxidation, a significant factor in food spoilage. According to the United Nations Food and Agriculture Organization (FAO), approximately one third of all food produced for human consumption worldwide is either spoiled or wasted, resulting in significant economic losses to the food industry. In the context of meat and meat products, lipid oxidation emerges as the principal non-microbial cause of quality degradation [1,2]. Similarly, in fruits and vegetables, oxidative processes lead to enzymatic and non-enzymatic browning, significantly impairing their sensory attributes, texture, and nutritional value [3]. The impact of oxidation extends beyond food, playing a pivotal role in human physiology. Oxidative cellular metabolism generates reactive oxygen species (ROS) [4], which are associated with the development of oxidative stress [5]. Prolonged oxidative stress has been implicated as a contributing factor to various diseases, including inflammatory disorders, cardiovascular diseases, diabetes mellitus, certain forms of cancer, and neurodegenerative conditions such as Alzheimer’s disease [6,7,8,9]. As a result, the World Health Organization has advocated for a worldwide increase in dietary antioxidants, as food intake is the main source of these compounds [10].

Synthetic antioxidants offer significant economic advantages and are highly effective. However, they are associated with specific toxicity and harmful effects. For example, the antioxidant BHA has been shown to lead to a higher incidence of forestomachal papilloma and squamous cell carcinoma [11]. In contrast, antioxidant peptides (AOPs) exhibit lower toxicity levels and are deemed safer for use as natural antioxidants. Notably, AOPs serve dual functions: on the one hand, they can prolong the shelf life of foods by preventing lipid oxidation processes; on the other hand, they mitigate oxidative stress in cells by neutralizing harmful free radicals [12]. To date, thousands of antioxidant peptides have been identified and extracted from various sources, such as meat products [13], seafood [14], plants [15], grains, and dairy products [16]. Despite the numerous advantages of AOPs, their identification primarily relies on conventional wet laboratory experiments. However, these experimental methods are labor intensive and time consuming, leading to inefficiencies. Consequently, there has been a growing interest in exploring advanced computational tools—especially artificial intelligence methods [17]—to predict antioxidant peptides more efficiently.

In recent years, several artificial intelligence methods have been developed to predict antioxidant peptides (AOPs). In 2020, T.H. Olsen et al. developed the first online server, AnOxPePred [18], an innovative online platform capable of leveraging neural networks for AOP prediction. This system utilizes one-hot encoding as the input feature vector and employs a convolutional neural network (CNN)-based framework. In 2022, Shen Yong et al. advanced the PseAAC [19], which involves pseudo amino acid composition (PseAAC) and motif-based feature extraction to predict AOPs. Although their study demonstrated promising results, it did not include comparative analyses against other approaches. In 2023, Qin Dongya et al. proposed the BiLSTM [20]-based AnOxPP [21,22], which demonstrated superior performance, with accuracies of 0.967 and 0.819 on two independent test sets, outperforming AnOxPePred. However, the large gap in indicators on the two test sets suggests limitations in robustness. In the same year, Du Zhenjiao et al. proposed the UniDL4BioPep [23] for predicting bioactive peptides. This framework utilized a transformer-based ESM-2 language model to generate fixed-length (320-dimensional) peptide sequence embeddings. In 2025, Li Wanxing et al. introduced a new BiLSTM-based model, AOPP [24], which surpassed AnOxPePred and AnOxPP in indicators on two different datasets and achieved state-of-the-art (SOTA) levels.

Although previous studies have achieved commendable performance in testing, notable limitations and shortcomings still remain. Regarding feature extraction, current research has predominantly focused on either sequence fingerprints or sequence evolution features extracted in a case-specific manner, thereby neglecting the global physicochemical property information of peptides. Furthermore, some studies focused solely on sequence fingerprints and physicochemical features while omitting sequence evolution features. In other domains, such as antimicrobial peptide research [25], models that integrate sequence fingerprints, sequence evolution features, and physicochemical property features have emerged [26,27,28,29], demonstrating performance far superior to approaches relying on single feature categories. This underscores the importance of combining diverse classes of features for accurately predicting peptide properties.

**Figure 1:**
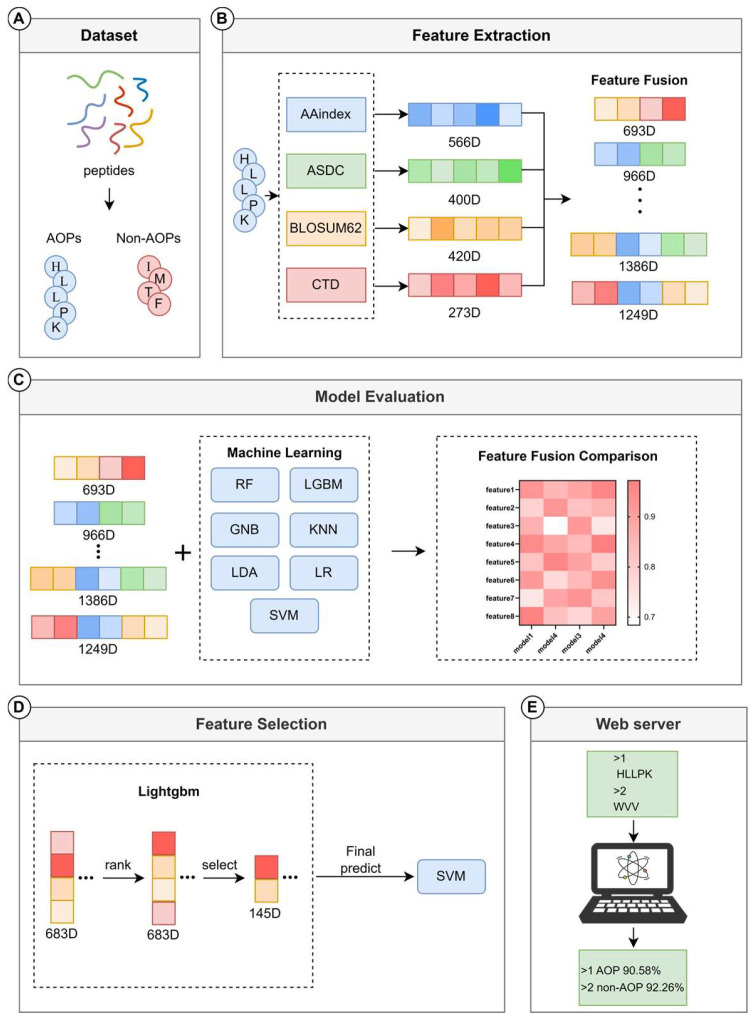
Technical flowchart. (**A**) Construction of the benchmark dataset. (**B**) Feature extraction using AAindex, ASDC, BLOSUM62, and CTD, which generated 566D AAindex features, 400D ASDC features, 420D BLOSUM62 features, and 273D CTD features, respectively. The 4 features were combined to generate 11 fusion features. (**C**) Generation of 90 different models by combining 15 different features (including single features) and 6 machine learning algorithms. (**D**) Feature selection for the best fusion feature, with the simplified feature inputted into the SVM to generate the final model. (**E**) Development of a web server based on the final optimized model.

In response to these limitations, this study proposed a new machine learning model: AOPxSVM. The construction process of AOPxSVM is shown in Figure 1. We introduced feature encoding methods such as ASDC, BLOSUM62, AAindex, and CTD, which represent three types of features: sequence fingerprints, sequence evolution features, and physicochemical property features. By integrating these features, we demonstrated the contribution of the complementarity of multidimensional information to the improvement in model performance. Then, we further optimized the feature vector using feature selection methods. Finally, we used the unified meteoroid approximation and projection (UMAP) algorithm for visualization to prove the effectiveness of feature engineering optimization. Compared with the existing optimal model, AOPP, AOPxSVM achieved significant improvements in independent test indicators on both datasets, demonstrating the advanced nature of this method. We expect that this study will help promote the application of machine learning models to predict antioxidant peptides in the food industry and ultimately promote the development of peptide-related research and industrial applications.

## 2. Materials and Methods

### 2.1. Benchmark Dataset

Our model was developed utilizing the latest AOPP dataset to facilitate the comparison with other models [24]. AOPP has two different datasets. The positive samples of the first dataset come from 1511 non-repeated AOPs from DFBP, the BI-OPEP-UWM database, the antimicrobial peptide database, and PlantPepDB. Peptide sequences were generated using a Python program. CD-HIT was used to filter out peptide sequences with a similarity of more than 90% with positive samples; then, an equal number of peptide sequences with the same length as the positive samples were randomly extracted to obtain the negative samples of the first dataset. The integrated dataset contains 3022 samples (1511 AOPs and 1511 non-AOPs), which were randomly divided into training and test sets at a ratio of 8:2. After this division, approximately 2417 samples were assigned to the training set and 604 samples were assigned to the test set. In addition, AOPP compiled an independent validation dataset of 75 peptide sequences with high antioxidant activity from the scientific literature from 2022 to 2023 to evaluate the performance of the model relative to existing research results. To clearly distinguish between the datasets in this study, the two datasets were termed AOPP.test01 and AOPP.test2023, respectively. The former was used to develop and improve the model, while the latter provided a benchmark for comparative performance evaluation. The details are shown in Table S1.

### 2.2. Feature Extraction

#### 2.2.1. Physicochemical Property Feature

(1) Amino Acid Index (AAindex)

The AAindex database [30] contains 566 physicochemical properties of amino acids, including measures such as hydrophobicity and polarity. A feature vector of dimensionality 566 was computed by calculating a weighted average of these physicochemical properties for each amino acid in the sequence.

(1) $$\begin{array}{c} V_{i}=\left(v_{1},v_{2},\ldots ,v_{566}\right) \end{array}$$

(2) $$\begin{array}{c} F=\frac{1}{L}\sum_{i=1}^{L}V_{i} \end{array}$$

where $v_{j}$ corresponds to the value of the *j*th physicochemical property of the amino acid, and $V_{i}$ corresponds to the vector of the *i*th amino acid in the peptide. *F* is the average value of each physicochemical property over all amino acids.

(2) Composition Transformation and Distribution (CTD)

CTD [31,32] systematically categorizes 20 amino acids into 3 distinct groups based on specific physicochemical properties. This classification enables the analysis of amino acid sequences through three primary components: composition (C)—this element quantifies the relative proportion of each amino acid group within the sequence; transition frequency (T), wherein the frequency of transitions between different amino acid groups is measured as they appear in the sequence; and distribution (D)—this component captures the positional distribution of amino acids through 5 specified quantiles: 0%, 25%, 50%, 75%, and 100%. These quantiles represent cumulative proportions across the sequence’s length at these intervals [33,34]. In this study, the CTD method incorporates 13 distinct physicochemical properties for classification purposes. These include characteristics such as hydrophobicity, polarizability, and charge, among others, ensuring a comprehensive analysis of amino acid traits. This process results in the generation of a feature vector characterized by 273 dimensions.

(3) $$\begin{array}{c} C_{i}=\frac{N_{i}}{L} \end{array}$$

(4) $$\begin{array}{c} T_{ij}=\frac{N_{ij}+N_{ji}}{L-1} \end{array}$$

(5) $$\begin{array}{c} D_{ik}=\frac{N_{ik}}{N_{i}} \end{array}$$

where *L* is the total length of the peptide sequence, $N_{i}$ is the number of amino acids in group *i*, $N_{ij}$ is the frequency of amino acid transitions between different groups, and $N_{ik}$ is the number of amino acids in group *i* within the first *k*% positions.

#### 2.2.2. Sequence Fingerprinting

(1) Adaptive Skip Dipeptide Composition (ASDC)

ASDC [35,36] captures amino acid associations at arbitrary distances in a sequence by counting the frequency of dipeptide combinations at all possible intervals in the peptide sequence. Its feature vector can be expressed as

(6) $$\begin{array}{c} ASDC=\left({fv}_{1,1},{fv}_{1,2},\ldots ,{fv}_{20,20}\right) \end{array}$$

where ${fv}_{i,j}$ represents the probability of each amino acid pair, with 400 amino acid pairs in total.

#### 2.2.3. Sequence Evolution Features

(1) Block Substitution Matrix 62 (BLOSUM62)

BLOSUM-n is a 20 × 20 amino acid substitution score matrix [37] designed to evaluate the likelihood of specific amino acid substitutions. BLOSUM-n calculates the substitution score s for two amino acids x and y. The calculation is as follows:

(7) sx,y=1λ log⁡pxyfxfy

where $p_{xy}$ is the target frequency, representing the probability of observing *x* and *y* aligned in the homologous sequence; and $f_{x},f_{y}$ are background frequencies that represent the probability of x and y occurring independently in the protein sequence. λ is a scaling factor used to change the score *s* to an integer, and *n* is the similarity of homologous sequences.

For peptide prediction purposes, we extended the BLOSUM62 matrix by appending a column of zero vectors, resulting in a 20 × 21 matrix. Subsequently, this expanded matrix was augmented with weights derived from amino acid frequencies in peptide sequences. This comprehensive approach yielded a 420-dimensional feature vector, capturing extensive amino acid substitution information for predictive modeling.

#### 2.2.4. Deep Learning-Based Embedded Features

(1) TAPE_BERT

BERT [38,39] is a transformer-based pre-training framework that performs self-supervised learning on unlabeled datasets to derive initial parameter values [40]. TAPE_BERT is trained on the Protein Family Database (Pfam) [41]. TAPE_BERT produces a 768-dimensional feature vector. The key formulation of the BERT model is as follows:

(8) $$\begin{array}{c} Attention\left(Q,K,V\right)=\mathrm{softmax}\left(\frac{QK^{T}}{\sqrt{d_{k}}}\right)V \end{array}$$

where *Q* is the query matrix, *K* is the key matrix, *V* is the value matrix, and $d_{k}$ is the vector dimension.

(2) UniRep

UniRep [42] is constructed based on a Multiplicative Long Short-term Memory (mLSTM), and unsupervised learning is performed on the UniRef50 [43] database containing 24 million proteins. The final feature vector of UniRep is a 1900-dimensional mLSTM network that can be defined by the following equation:

(9) $$\begin{array}{c} m_{t}=\left(W_{mx}x_{t}\right)⊙\left(W_{mh}h_{t-1}\right) \end{array}$$

(10) $$\begin{array}{c} {\hat{h}}_{t}=W_{hx}x_{t}+W_{hm}m_{t} \end{array}$$

(11) $$\begin{array}{c} i_{t}=\sigma \left(W_{ix}x_{t}+W_{im}m_{t}\right) \end{array}$$

(12) $$\begin{array}{c} o_{t}=\sigma \left(W_{ox}x_{t}+W_{om}m_{t}\right) \end{array}$$

(13) $$\begin{array}{c} f_{t}=\sigma \left(W_{fx}x_{t}+W_{fm}m_{t}\right) \end{array}$$

(14) $$\begin{array}{c} c_{t}=f_{t}⊙c_{t-1}+i_{t}⊙tanh\left(h_{t}\right) \end{array}$$

(15) $$\begin{array}{c} h_{t}=tanh\left(c_{t}\right)⊙o_{t} \end{array}$$

where $m_{t}$ is the current multiplication interim state, *W* denotes the weight matrix, $x_{t}$ is the current time step input, $h_{t-1}$ is the previous time step hidden state, $i_{t},o_{t},f_{t}$ stand for the input gate, output gate, and forgetting gate, respectively, and σ is the sigmoid activation function. $c_{t}$ is the state of the memory cell, which is used to update the long-term memory.

### 2.3. Machine Learning Methods

In the context of this study, we employed six widely recognized machine learning (ML) methodologies, each distinguished for its performance and applicability across diverse circumstances [44]. These include Support Vector Machine (SVM) [45,46,47,48], Light Gradient Boosting Machine (LGBM) [49], Linear Regression (LR) [50], Random Forest (RF) [51,52], K-Nearest Neighbors (KNN) [53], and Gaussian Naive Bayes (GNB) [54].

The objective of the SVM algorithm is to find a hyperplane to maximize the interval between different classes of data and map the data to a high dimensional space utilizing a kernel function. SVM also solves nonlinearly differentiable problems and, thus, can be used to solve binary classification problems in bioinformatics.

LGBM is a decision tree-based algorithm that employs a gradient boosting strategy to optimize the model.

LR solves the classification problem by mapping the linear output to the interval [0, 1] using a sigmoid function, which represents the probability that the sample belongs to a particular class.

RF is an integrated learning algorithm that decides the outcome by training multiple decision trees and voting.

KNN is an instance-based learning algorithm that classifies samples by calculating the distance between them; when a new sample is input, the class of the new sample is determined by voting on the K closest sample points.

GNB is a classification algorithm based on the Bayes theorem.

### 2.4. Feature Selection Methods

Feature selection is essential for developing robust predictive models [55]. This process effectively reduces dimensionality by eliminating redundant features while preserving critical ones, thereby enhancing model interpretability and performance. LGBM (Light Gradient Boosting Machine), a powerful gradient-boosting decision tree framework [56], determines feature importance by quantifying the number of splits each feature undergoes in the trees, subsequently ranking them in descending order. Previous studies have shown that LGBM outperforms both analysis of variance (ANOVA) [57] and mutual information (MI) [58] in feature selection for peptide sequences [50]. The mathematical formula for LGBM feature importance ranking is as follows:

(16) $$\begin{array}{c} Importance_{\mathrm{split}}^{f}=\sum_{t=1}^{T}\sum_{n=1}^{N_{t}}I\left(v\left(t,n\right)=f\right) \end{array}$$

where $Importance_{\mathrm{split}}^{f}$ is the splitting importance of the feature, *T* is the total number of trees, $N_{t}$ is the number of nodes in the *t*-th tree, $v\left(t,n\right)$ is the feature used by the *n*-th node in the *t*-th tree, and *I* () is the indicator function, which is 1 if the condition is met; otherwise, it is 0.

### 2.5. Model Evaluation Metrics

To evaluate the model’s performance, we used seven assessment metrics, including accuracy (ACC), Matthew’s correlation coefficient (MCC), sensitivity (Sn), specificity (Sp), precision (Pre), area under the curve (AUC), and F1 score. These indicators were calculated from the numbers of true-positive samples (TP), true-negative samples (TN), false-positive samples (FP), and false-negative samples (FN) [59,60,61,62,63,64,65].

(17) $$\begin{array}{c} ACC=\frac{TP+TN}{TP+TN+FP+FN} \end{array}$$

(18) $$\begin{array}{c} MCC\;=\;\frac{TP\;\times \;TN\;-\;TP\;\times \;TN}{\sqrt{\left(TP\;+\;FP\right)\left(TP\;+\;TN\right)\left(TN\;+\;TP\right)\left(TN\;+\;FN\right)}} \end{array}$$

(19) $$\begin{array}{c} Sn=\frac{TP}{TP+FN} \end{array}$$

(20) $$\begin{array}{c} Sp=\frac{TN}{TN+FP} \end{array}$$

(21) $$\begin{array}{c} Pre=\frac{TP}{TP+FP} \end{array}$$

(22) $$\begin{array}{c} F1=\frac{2\times Pre\times Sn}{Pre+Sn} \end{array}$$

Among them, the F1 score represents the harmonic mean of precision and recall, which comprehensively considers the accuracy and completeness of positive class predictions and is particularly suitable for situations where positive and negative samples are unevenly distributed. MCC, as a balance indicator, can comprehensively consider the number of TP, TN, FP, and FN, and is considered the gold standard for measuring the performance of binary classification models. Finally, AUC is the area under the ROC curve, which reflects the model’s ability to distinguish between positive and negative samples under all possible thresholds.

### 2.6. Friedman Test

The Friedman test [41] is a nonparametric statistical test applied to random area groups proposed by Milton Friedman. It avoids the reliance on the assumption of a normal distribution of data in the traditional analysis of variance (ANOVA). The Friedman test sorts the samples into blocks, sums up the sorts of all the treatment groups, and statistically analyzes them by the difference between the sorted sums. The formula for the Friedman test is as follows:

(23) $$\begin{array}{c} \chi_{F}^{2}=\frac{12}{Nk\left(k+1\right)}\sum_{j=1}^{k}R_{j}^{2}-3N\left(k+1\right) \end{array}$$

where *N* is the number of blocks (number of different machine learning metrics), *k* is the number of treatment groups (number of different features or models), and *R_j_* is the total rank sum of the *j*th treatment group.

## 3. Results

### 3.1. Selection of Baseline Models with Different Features and Fusion Features

**Figure 2:**
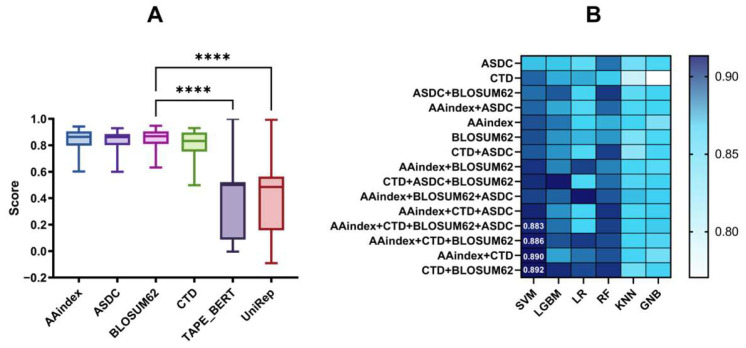
Comparison of machine learning metrics for different feature encoding methods. (**A**) Comparison of machine learning metrics with 6 different single features (score is the score of 6 independently tested metrics: ACC, MCC, Sn, Sp, AUC, and Pre. The boxplot uses Friedman’s statistical test, and the labeled * denotes the significance of the statistical test, where **** represents *p* < 0.0001, and ns represents no statistical significance). (**B**) Comparison of the 15 features with 6 machine learning combinations of independently tested metrics (the 4 highest-scoring features in SVM are labeled).

We evaluated 36 baseline models that combined 6 features with 6 machine learning algorithms to determine the optimal feature extraction method. As summarized in Table S2, these models underwent a comparative analysis based on their five-fold cross-validation accuracy. Among the features examined, ASDC demonstrated superior performance across multiple algorithms, achieving the highest accuracy scores of 0.8920 (SVM), 0.8891 (RF), 0.8849 (LGBM), and 0.8634 (GNB). Conversely, AAindex and BLOSUM62 exhibited the strongest performance for KNN (0.8398) and LR (0.8713), respectively. The comparative performance of these features on an independent test set is illustrated in Figure 2, with statistical significance analyzed using Friedman’s test. The findings reveal that TAPE_BERT and UniRep exhibited significantly weaker performance compared with the remaining four features (*p* < 0.0001). In contrast, AAindex, ASDC, BLOSUM62, and CTD displayed no statistically significant differences in their performance outcomes (*p* > 0.05). Based on this analysis, we proceeded with these four features for subsequent optimization in the feature fusion process.

Combining multiple features enhances information complementarity and improves the robustness of predictive models. In this study, we developed 11 distinct fusion features by integrating AAindex, ASDC, BLOSUM62, and CTD features. As shown in Table S3, SVM achieved the highest scores across five metrics among all six machine learning algorithms, except for specificity (Sp). Figure 2B presents a comparative analysis of the scores for 90 models incorporating these fusion features and the 6 machine learning algorithms. The scores represent the averages across six independently evaluated metrics, with labels indicating the four top-performing feature combinations in SVM. Among these fusion features, the CTD + BLOSUM62 fusion feature demonstrated the most superior performance. Compared with the best-performing single features, this combination achieved significant improvements; the ACC on the independent test increased by 5.08%, the MCC by 10.57%, the Sn by 1.52%, the Sp by 4.09%, the AUC by 4.08%, and the Pre by 6.30%. Therefore, we chose the SVM model paired with the CTD + BLOSUM62 fusion feature for subsequent optimization steps. Meanwhile, these results demonstrate that fusion features significantly enhance the predictive capabilities of the model compared with single-feature approaches.

### 3.2. Feature Selection Optimization

**Figure 3:**
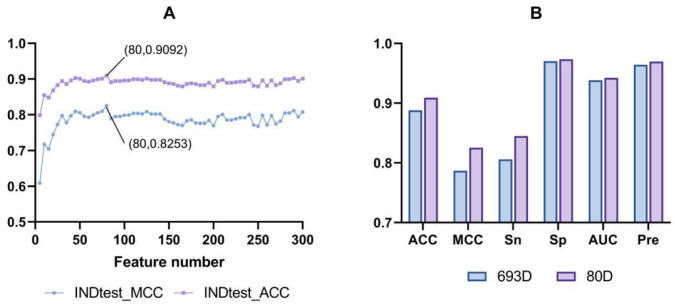
(**A**) Changes in ACC and MCC during feature selection of CTD + BLOSUM62. (Best model Params{‘C’: 2.782559402207126, ‘gamma’: 0.005994842503189409, ‘kernel’: ‘rbf’}). (**B**) Comparison of independent test indicators of the model before and after feature selection.

High-dimensional features often lead to redundancy and overfitting. To address this, we implemented a feature selection strategy to reduce dimensionality while enhancing model performance. Using the built-in function in LGBM, we ranked the features by counting the number of splits in the trees, reflecting their importance. With a step size of 5, we selected the top 5, 10, …, 295, and 300 features iteratively, building an SVM-based model at each interval. The optimization process outlined in Figure 3A reveals that the ACC and MCC initially increased rapidly, peaked, declined, and then stabilized. Both ACC and MCC reached their highest values at a feature count of 80, achieving values of 0.9092 and 0.8253, respectively. As shown in Figure 3B, comparing six independent test metrics before and after selecting the CTD + BLOSUM62 features indicates significant improvement across all criteria. Notably, ACC, MCC, and Sn showed the most substantial enhancement. Consequently, we chose the optimized 80D CTD + BLOSUM62 feature set to construct our final SVM-based model, AOPxSVM, for subsequent analyses.

### 3.3. Feature Visualization

**Figure 4:**
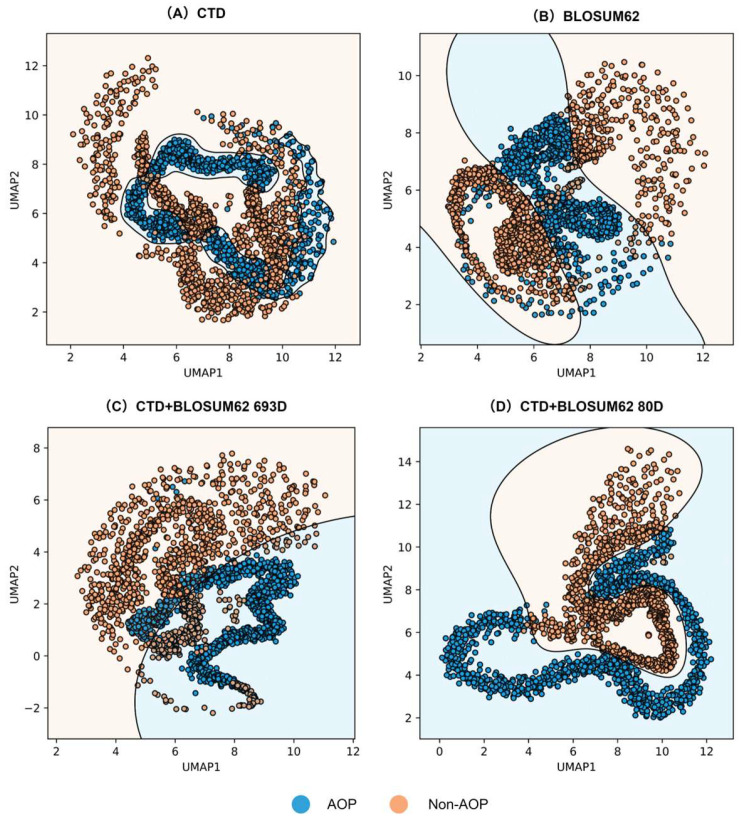
Visualization of four features using UMAP dimensionality reduction. (parameters: {‘metric’: ‘wminkowski’, ‘n_neighbors’: 10, ‘min_dist’: 0.2, ‘target_weight’:’0.2’}) (**A**) CTD feature; (**B**) BLOSUM62 feature; (**C**) CTD + BLOSUM62_693D feature; (**D**) CTD + BLOSUM62_125D feature.

We applied UMAP for dimensionality reduction to 2D and visualized the SVM decision boundaries based on these projections to validate the effectiveness of the feature engineering optimization. As shown in Figure 4A–C, feature fusion results in better clustering than single features alone. Furthermore, as demonstrated in Figure 4C vs. Figure 4D, the selection process yields improved clustering over unselected features. These findings confirm that the feature engineering optimization successfully eliminated redundant features from fused sets and enhanced the discrimination between antioxidant peptide and non-antioxidant peptide features.

### 3.4. Comparison with Existing Methods

To evaluate the performance of our AOPxSVM model against existing methods, we compared two independent test sets. First, on the AOPP.test01 dataset, we compared AOPxSVM with AOPP, the current state-of-the-art method. As shown in Table 1, AOPxSVM demonstrates superior performance across multiple metrics: Val_ACC (0.9056), ACC (0.9092), MCC (0.8253), Sn (0.8449), AUC (0.9423), and F1 (0.9030) all outperform those of AOPP. To ensure a fair comparison, we also evaluated the models on the AOPP.test2023 test set, which contains 75 recently identified antioxidant peptides from the literature (2022–2023). Notably, this dataset is completely different from the training set of our model and AOPP.test01. The results presented in Table 1 further highlight the superiority of AOPxSVM across key metrics: ACC is 0.66% higher, MCC is 0.75% higher, Sn is 5.33% higher, and F1 is 1.04% higher compared with AOPP. These findings confirm that AOPxSVM achieves the best performance on both independent test sets, underscoring the model’s generalizability and robustness.

To further test the generalization of the model, we collected 138 new antioxidant peptide sequences from the recent year’s literature as an independent test set, which are completely different from the previous two datasets. In order to control data redundancy, we used the CD-HIT tool to perform de-redundancy processing at sequence similarity thresholds of 40%, 60%, and 80%, respectively, to obtain datasets with different similarity levels. As shown in Figure S1, AOPxSVM showed the highest ACC at all three similarity levels, further demonstrating the superiority of AOPxSVM.

### 3.5. Web Server Development

To facilitate a researcher’s use of our model to predict antioxidant peptides, we deployed the model to a web server, which can be accessed via http://inova.aibiochem.net/antioxpep/ (accessed on 3 June 2025). Users can obtain information about the antioxidant activity and confidence of samples by inputting peptide sequences or fasta files.

## 4. Discussion

The prediction of antioxidant peptides holds significant importance for the food industry, and many methods are available for antioxidant peptide prediction and screening. However, the existing approaches still face challenges related to accuracy and robustness, largely due to inadequate optimization for diverse feature types. Such comprehensive optimization is critical for enhancing the reliability and interpretability of predictive models. To address these limitations, we developed a novel AOP prediction model, AOPxSVM, utilizing CTD + BLOSUM62 fusion features. This model demonstrates superior performance compared with existing methods on two independent test sets, suggesting its potential to further advance the field of antioxidant peptide prediction.

In this study, we first evaluated the fusion of four different features, among which the fusion of the physicochemical property feature CTD and the sequence evolution feature BLOSUM62 obtained the best results. This finding aligns with research results reported by researchers at the China Agricultural University in 2025, who identified correlations between the antioxidant properties of fish gelatin peptides and both global electron donor capacity (a physicochemical property) and local active sites (a sequence evolutionary feature), as determined through quantum computing (DFT) and molecular docking [66]. This agreement underscores our conclusion that combining physicochemical and sequence evolution features may better capture the characteristics of antioxidant peptides. Second, we used feature selection means to optimize our fusion features, which resulted in a dimension of only 80 D. In contrast, AOPP used the feature ADCA (spliced with AAC, DPC, CKS, and AAindex) with a dimension of more than 800 D. This suggests that our model has stronger interpretability, which is a major challenge in machine learning [67,68]. We further emphasize this with our UMAP visualization.

Despite these advancements, certain limitations remain. First, the development of high-quality, large-scale datasets remains crucial for improving model accuracy and robustness [69,70]. Additionally, due to the constraints inherent in traditional machine learning approaches, we were unable to retain sequence-specific information during training; instead, our framework relied solely on global features extracted from the data. This omission may have impacted the performance of our fused feature set. In future work, we aim to address these limitations by integrating a dual-path model that combines machine learning and deep learning. Such an approach would enable separate processing of global and sequence-specific features, potentially leading to more robust models capable of capturing the full breadth of feature information. This will be a key focus of our subsequent research efforts.

## 5. Conclusions

In this study, we developed a model called AOPxSVM to predict antioxidant peptides, which are essential compounds with significant potential in the food industry. The process involved selecting feature extraction methods from an initial pool of six approaches. Through rigorous evaluation, we identified four superior methods: AAindex, ASDC, CTD, and BLOSUM62. These selected features were integrated with six machine learning algorithms to optimize predictive performance. Our analysis revealed that the SVM algorithm demonstrated the highest efficacy for this classification task. The optimal fusion features were CTD, representing physicochemical properties, and BLOSUM62, capturing sequence evolution characteristics. To enhance computational efficiency and reduce overfitting risks, we implemented feature selection techniques, reducing the feature count from 693D to a more manageable 80D. Finally, we obtained the final model AOPxSVM. We also employed UMAP visualization to demonstrate the effectiveness of our feature engineering approach. On two different independent test sets, our model AOPxSVM obtained the best results, surpassing those of other models in ACC, MCC, Sn, Pre, and F1, with ACCs reaching 0.9092 and 0.9333, and MCCs reaching 0.8253 and 0.8670, respectively. In conclusion, based on feature engineering optimization and machine learning algorithms, our study proposed an accurate and reliable binary classification of antioxidant peptides. This methodological framework enhances predictive capabilities and provides a robust foundation for future applications in bioinformatics and food science, contributing to the development of innovative strategies for leveraging antioxidant peptides in industrial settings.

## Figures and Tables

**Table 1:** Comparison of indicators between AOPxSVM and existing models.

Test Dataset	AOPP.test01								AOPP.test2023					
Model	Val_ACC	ACC	MCC	Sn	Sp	AUC	Pre	F1	ACC	MCC	Sn	Sp	Pre	F1
AOPP	0.8969	0.9043	0.8181	0.8284	0.9802	0.9043	0.9767	0.8965	0.9267	0.8595	0.8667	0.9867	0.9848	0.9220
AnOxPP	——	——	——	——	——	——	——	——	0.8800	0.7610	0.9060	0.8530	0.8610	0.8829
AnOxPePred ^a^	——	——	——	——	——	——	——	——	0.7530	0.4330	0.8100	0.6270	0.8260	0.8179
UniDL4BioPep	——	——	——	——	——	——	——	——	0.5800	0.1633	0.6800	0.4800	0.5667	0.6182
SBSM-Pro	——	0.7888	0.5786	0.7591	0.8185	——	0.8070	0.7823	0.7333	0.4668	0.7200	0.7467	0.7397	0.7297
AOPxSVM *	0.9056	0.9092	0.8253	0.8449	0.9736	0.9423	0.9697	0.9030	0.9333	0.8670	0.9200	0.9467	0.9452	0.9324

Note: * indicates the model of this study, and ^a^ indicates the model result of the model tested using its own dataset. The best value in each column is shown in underlined.

## Data Availability

The original contributions presented in this study are included in the article/Supplementary Materials. The source code is available at https://github.com/yashdui/AOPxSVM.git (accessed on 3 June 2025). Further inquiries can be directed to the corresponding author.

## Supplementary Materials

The following supporting information can be downloaded at https://www.mdpi.com/article/10.3390/foods14122014/s1, Table S1: Composition of all datasets; Table S2: Comparison of 5-fold cross-validation accuracy of 6 features in 6 machine learning algorithms; Table S3: Independent test indicators of 6 machine learning algorithms (scores are the average of 15 features); Table S4: Comparison of 5-fold cross-validation and independent test indicators based on the SVM model after feature fusion; Table S5: Comparison of independent test results of four fusion features after feature selection; Table S6: Hyperparameter ranges and optimization strategy; Figure S1: Comparison of models’ ACC at different sequence similarity thresholds.
