# Supplementary material for: AOPxSVM: A Support Vector Machine for Identifying Antioxidant Peptides Using a Block Substitution Matrix and Amino Acid Composition, Transformation, and Distribution Embeddings
Source: Foods. 2025 Jun 6;14(12):2014. doi: 10.3390/foods14122014 (PMC12192177; doi:10.3390/foods14122014)
Supplement: Supplementary file 1 [file foods-14-02014-s001.zip › foods-3616141-supplementary.pdf]

Table S1. Composition of all datasets

Dataset	Postive	Negative
**AOPP**	1511	1511
**Train Dataset**	1208	1208
**AOPP.test01**	303	303
**AOPP.test2023**	75	75

Table S2. Comparison of 5-fold cross-validation accuracy of 6 features in 6 machine learning algorithms.

**Metrics**	**5-Fold Cross-Validation_ACC**					
**Features/Algorithms**	**SVM**	**RF**	**LR**	**LGBM**	**KNN**	**GNB**
**AAindex**	0.8829	0.8725	0.8667	0.8816	**0.8398**	0.8175
**ASDC**	**0.8920**	**0.8891**	0.8398	**0.8849**	0.7930	**0.8634**
**BLOSUM62**	0.8887	0.8642	**0.8713**	0.8837	0.8361	0.8514
**CTD**	0.8775	0.8560	0.8642	0.8742	0.7682	0.7794
**TAPE_BERT**	0.7901	0.7595	0.8224	0.8539	0.8199	0.8228
**UniRep**	0.7347	0.7653	0.7285	0.7860	0.7765	0.7926

Table S3. Independent test indicators of 6 machine learning algorithms (scores are the average of 15 features)

Features/Metrics	ACC	MCC	Sn	Sp	AUC	pre
**SVM**	**0.8776**	**0.7612**	0.8168	**0.9384**	**0.9321**	**0.9305**
**LGBM**	0.8705	0.7446	0.8234	0.9177	0.9273	0.9095
**LR**	0.8597	0.7214	**0.8243**	0.8951	0.9150	0.8876
**RF**	0.8733	0.7507	0.8230	0.9237	0.9284	0.9154
**KNN**	0.8174	0.6440	0.7379	0.8969	0.8828	0.8785
**GNB**	0.8304	0.6674	0.7674	0.8934	0.8716	0.8792

Table S4. Comparison of 5-fold cross-validation and independent test indicators based on SVM model after feature fusion

Features	5-Fold Cross-Validation						Independent Test					
	ACC	MCC	Sn	Sp	AUC	Pre	ACC	MCC	Sn	Sp	AUC	Pre
**AAindex**	0.8829	0.7696	0.8361	0.9296	0.9442	0.9226	0.8373	0.6812	0.7683	0.9063	0.8976	0.8914
**ASDC**	0.8920	0.7862	**0.8593**	0.9247	0.9460	0.9200	0.7875	0.5996	0.6455	0.9294	0.8678	0.9014
**BLOSUM62**	0.8887	0.7818	0.8361	0.9412	0.9421	0.9345	0.8155	0.6318	0.7908	0.8403	0.8766	0.8320
**CTD**	0.8775	0.7593	0.8262	0.9288	0.9383	0.9209	0.7667	0.5397	0.6904	0.8429	0.8330	0.8146
**AAindex+CTD**	0.8812	0.7643	0.8527	0.9098	0.9419	0.9551	0.8878	0.7841	**0.8145**	0.9611	0.9382	0.9544
**CTD,BLOSUM62**	**0.8998**	**0.8049**	0.8452	**0.9545**	**0.9488**	**0.9626**	**0.8881**	**0.7869**	0.8059	**0.9703**	**0.9384**	**0.9645**
**AAindex,CTD,BLOSUM62**	0.8808	0.7632	0.8501	0.9114	0.9459	0.9578	0.8842	0.7770	0.8099	0.9584	0.9382	0.9513
**AAindex,CTD,BLOSUM62,ASDC**	0.8622	0.7246	0.8651	0.8593	0.9245	0.9390	0.8828	0.7725	0.8165	0.9492	0.9346	0.9414

Table S5. Comparison of independent test results of four fusion features after feature selection

Features	ACC	MCC	Sn	Sp	AUC	pre
**AAindex+CTD**	0.9026	0.8114	0.8416	0.9637	0.9430	0.9586
**CTD+BLOSUM62**	**0.9092**	**0.8253**	0.8449	**0.9736**	0.9423	**0.9697**
**AAindex+CTD+BLOSUM62**	**0.9092**	0.8240	0.8515	0.9670	0.9440	0.9627
**AA-index+CTD+BLOSUM62+ASDC**	0.9059	0.8151	**0.8614**	0.9505	**0.9442**	0.9457

Table S6. Hyperparameter Ranges and Optimization Strategy

Model	Hyperparameters	Search Range	Optimization Method
**SVM**	C, gamma, kernel	C: logspace(-4, 4, 10)<br>gamma: logspace(-4, 4, 10)<br>kernel: 'rbf'	GridSearchCV*
**GNB**	var_smoothing	var_smoothing:[10e-9]	GridSearchCV
**RF**	n_estimators,<br>criterion,<br>min_samples_leaf	n_estimators: 25 to 525 (step=25) crite-<br>rion: 'gini', 'entropy'<br>min_samples_leaf: 2 to 11	GridSearchCV
**LGBM**	n_estimators,<br>learning_rate,<br>max_depth	n_estimators: 100 to 800 (step=50)<br>learning_rate: 0.05, 0.01, 1<br>max_depth: 3, 6, 9, 12	GridSearchCV
**LR**	penalty, C	penalty: 'l1', 'l2', 'elasticnet', 'none'<br>C: logspace(-5, 5, 200)	GridSearchCV
**KNN**	n_neighbors	3, 5, 7, 9, 11	GridSearchCV

*All model hyperparameters were optimized using grid search. For each model, a predefined range of hyperparameters (as detailed in Table S6) was systematically explored. Model performance for each parameter combination was evaluated using stratified K-fold cross-validation (K=5). The optimal hyperparameter set was selected based on the highest cross-validated accuracy (refit='Accuracy'). To ensure robust model evaluation, additional performance metrics, including precision, recall, F1-score, and AUC, were also calculated and reported.

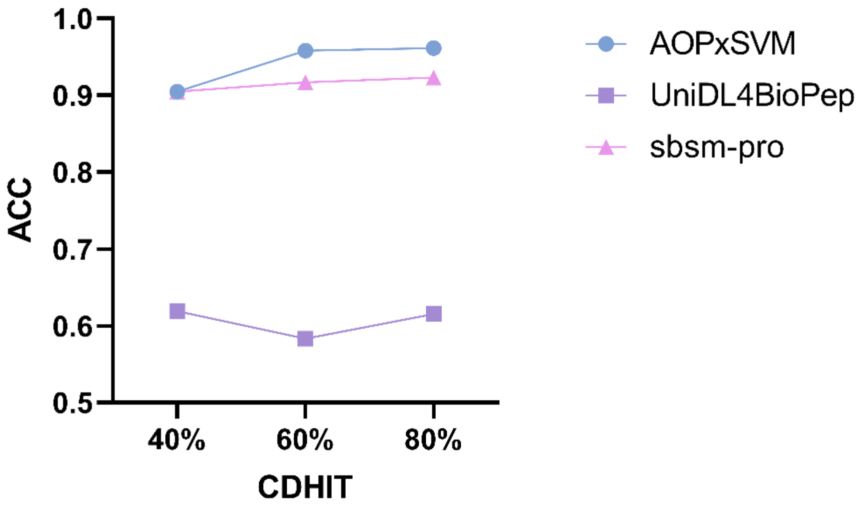

Figure S1. Comparison of Models' ACC at Different Sequence Similarity Thresholds

Our independent test set data are collected from various literature sources from past three years[1-7]. We used the CD-HIT tool and retained 21, 24, and 26 peptide sequences at 40%, 60%, and 80% similarity thresholds, respectively. The ACCs of AOPxSVM at 40%, 60%, and 80% similarity thresholds are 0.9048, 0.9583, and 0.9615, respectively, which are better than other models.

1. Guo, X.; Liu, J.; Wang, C.; Wen, Z.; Zheng, B. The antioxidant mechanism of peptides extracted from tuna protein revealed using a molecular docking simulation. *Antioxidants* **2024**, *13*, 166.
2. Hesamzadeh, P.; Seif, A.; Mahmoudzadeh, K.; Ganjali Koli, M.; Mostafazadeh, A.; Nayeri, K.; Mirjafary, Z.; Saeidian, H. De novo antioxidant peptide design via machine learning and DFT studies. *Scientific Reports* **2024**, *14*, 6473.
3. Li, W.; Liu, X.; Liu, Y.; Zheng, Z. High-Accuracy Identification and Structure–Activity Analysis of Antioxidant Peptides via Deep Learning and Quantum Chemistry. *J Chem Inf Model* **2025**, *65*, 603-612.
4. Liu, X.; Hu, Q.; Shen, Y.; Wu, Y.; Gao, L.; Xu, X.; Hao, G. Research Progress on Antioxidant Peptides from Fish By-Products: Purification, Identification, and Structure–Activity Relationship. *Metabolites* **2024**, *14*, 561.
5. Wang, Q.; Wang, L.; Huang, Z.; Xiao, Y.; Liu, M.; Liu, H.; Yu, Y.; Liang, M.; Luo, N.; Li, K. Abalone peptide increases stress resilience and cost-free longevity via SKN-1-governed transcriptional metabolic reprogramming in C. elegans. *Aging Cell* **2024**, *23*, e14046.
6. Wang, Y.; Zhao, B.; Ding, Y.; Liu, N.; Yang, C.; Sun, Y. Improved Anti-Oxidant and Anti-Bacterial Capacities of Skim Milk Fermented by Lactobacillus plantarum. *Molecules* **2024**, *29*, 3800.
7. Zhao, D.; Liu, X. Purification, identification and evaluation of antioxidant peptides from pea protein hydrolysates. *Molecules* **2023**, *28*, 2952.
